# Poor outcome after a surgically treated chondral injury on the medial femoral condyle: early evaluation with dGEMRIC and 17-year radiographic and clinical follow-up in 16 knees

**DOI:** 10.1080/17453674.2018.1481304

**Published:** 2018-06-05

**Authors:** Jon Tjörnstrand, Paul Neuman, Björn Lundin, Jonas Svensson, Leif E Dahlberg, Carl Johan Tiderius

**Affiliations:** 1 Department of Orthopaedics, Clinical Sciences, Lund, Lund University;; 2 Department of Orthopaedics, Clinical Sciences, Malmö, Lund University;; 3 Department of Radiology, Clinical Sciences, Lund, Lund University;; 4 Department of Medical Radiographic Physics, Clinical Sciences, Malmö, Lund University, Sweden

## Abstract

Background and purpose — The optimal treatment for traumatic cartilage injuries remains unknown. Contrast-enhanced MRI of cartilage (dGEMRIC) evaluates cartilage quality and a low dGEMRIC index may predict radiographic osteoarthritis (OA). The purpose of this study was (a) to explore the results 17 years after surgical treatment of an isolated cartilage knee injury and (b) to evaluate the predictive value of dGEMRIC.

Patients and methods — 16 knees with an isolated traumatic cartilage injury of the medial femoral condyle had cartilage repair surgery either by microfracture or autologous cartilage implantation. dGEMRIC of the injured knee was performed 2 years after surgery and radiographic examinations were performed 17 years after the operation.

Results — Radiographic OA was present in 12 of 16 knees. Irrespective of surgical method, the dGEMRIC index was lower in repair tissue compared with adjacent cartilage in the medial compartment, 237 ms vs. 312 ms (p < 0.001), which in turn had lower value than in the non-injured lateral cartilage, 312 ms vs. 354 ms (p < 0.008). The dGEMRIC index in the cartilage adjacent to the repair tissue correlated negatively with radiographic osteophyte score, r = –0.75 (p = 0.03).

Interpretation — A traumatic cartilage injury is associated with a high prevalence of OA after 17 years. The low dGEMRIC index in the repair tissue 2 years postoperatively indicates fibrocartilage of low quality. The negative correlation between the dGEMRIC index in the adjacent cartilage and future OA suggests that the quality of the surrounding cartilage influences outcome after cartilage repair surgery.

Cartilage injuries, with or without complicating ligamentous/meniscal injury, often occur after a twisting/compression trauma during sports activities. Cartilage has a limited healing potential with a complex structure with low chondrocyte density and avascularity. The best treatment for chondral defects remains controversial, despite decades of efforts (Hunziker et al. 2015). Microfracture (MFX) and autologous chondrocyte implantation (ACI) are the most commonly used techniques. In MFX, mesenchymal stem cells are recruited from the bone marrow by drilling or punching multiple holes in the subchondral bone plate of the cartilage lesion. First described in 1959 and having subsequently evolved, this is presently the most used technique (Pridie 1959, Steadman et al. 2001). ACI is a more technically demanding procedure, introduced in 1994 (Brittberg et al. 1994). This first-generation ACI includes two surgical procedures with arthroscopic harvesting of cartilage at the first operation for in vitro cultivation of chondrocytes. At the second operation, 3 weeks later, the expanded chondrocytes are injected under a periosteum flap that is sutured over the cartilage defect.

For both the MFX and the ACI technique, good 2–8 year results have been reported in younger patients with a well-defined traumatic injury. No clear difference has been observed between these treatments regarding failure rate or clinical outcome (PROMS) in randomized controlled trials (RCT) (Kraeutler et al. 2018). However, in a longer perspective, > 10 years, several studies have shown that approximately half of the patients have developed radiographic osteoarthritis (OA), after both MFX and ACI treatment (Gobbi et al. 2014, Martinčič et al. 2014, Knutsen et al. 2016).

OA is a slowly developing degenerative disease on the timescale of 10 to 20 years. In the very early stages of the disease, molecular and cellular processes decrease cartilage quality but symptoms or radiographic changes may not yet be present.

Delayed gadolinium-enhanced MRI of cartilage (dGEMRIC) is a non-invasive method to assess and monitor such early degenerative changes in vivo. The method is based on the principle that a negatively charged contrast agent distributes into articular cartilage in an inverse relationship to the concentration of negatively charged glucosaminoglycans (GAG). In several clinical studies, the method has demonstrated a predictive potential for subsequent OA development, both in the hip (Cunningham et al. 2006, Palmer et al. 2017) and the knee (Owman et al. 2008, 2014).

The aims of the present study were to: (a) study the radiographic progression to OA 17 years after surgical treatment of a traumatic chondral injury on the medial femoral condyle, and (b) to evaluate if dGEMRIC has a predictive value in terms of future OA development.

Patient data, dGEMRIC index at 27 months, and radiographic outcome at 17 years

**Table:** 

A	B	C	D	E	F	G	H	I
1	M	36	ACI	150	346	287	259	HTO at 3 years, presently scheduled for TKA
2	M	36	ACI	200	367	362	213	No radiographic OA, Clinical OA
3	M	37	ACI	300	334	315	253	< 2 years conversion to mosaic, HTO at
								16 years, presently considering TKA
4	F	37	MFX	300	343	245	241	Radiographic OA, clinical OA
5	M	45	MFX	400	311	243	218	Radiographic OA, clinical OA
6	F	36	ACI	225	330	370	249	UKA at 6 years
7	M	36	ACI	600	381	306	210	Radiographic OA, clinical OA
8	M	31	ACI	600	269	292	241	TKA at 14 years
9	F	37	MFX	200	446	306	276	UKA at 7 years
10	F	30	ACI	200	385	309	250	No radiographic OA, clinical OA
11	M	34	MFX	250	376	281	231	Radiographic OA at 9 years
12	F	39	MFX	150	382	297	216	Radiographic OA, clinical OA
13	F	40	ACI	150	271	332	263	TKA at 13 years
14	M	38	ACI	150	347	293	239	No radiographic OA, no OA symptoms
15	M	38	MFX	180	327	325	207	Radiographic OA at 4 years
16	M	30	ACI	120	453	428	235	No radiographic OA, no OA symptoms
Mean		37.2		261	354	312	237	
(SD)		(4.7)		(151)	(51)	(46)	(21)	

A Studied knee number

B Sex

C Age at index operation

D Cartilage repair procedure

E Size of lesion, mm^2^

F dGEMRIC index lateral at 27 months

G dGEMRIC index medial (adjacent to repair) at 27 months

H dGEMRIC index repair tissue at 27 months

I Outcome: radiographic OA, OA surgery, or clinical OA by KOOS score

Radiographic OA was defined using the OARSI score and clinical OA defined using the Knee Osteo­arthritis Outcome Score (KOOS). 1 patient had bilateral operations (knees number 12 and 13). 2 patients (knees number 11 and 15) did not participate in the 17-year radiographic follow-up but had radiographs recorded 4 and 9 years postoperatively. Knees that had undergone OA surgery (high tibial osteotomy (HTO), unicompartmental knee arthroplasty (UKA) or total knee arthroplasty (TKA)) were dichotomized as OA diagnosis but excluded from analysis of outcome measures and radiographic change.

## Patients and methods

### Patients

**Figure 1.**
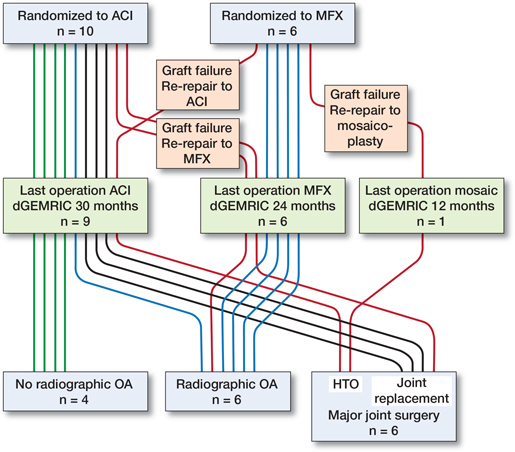
Flow-chart of treatment and follow-up of all 16 knees (in 15 patients).

Between 1997 and 2000, 16 knees in 15 patients were treated surgically due to a symptomatic isolated traumatic cartilage injury on the medial femoral condyle. Patients had no symptoms before the injury of the affected knee. The patients were initially included in an RCT of ACI vs. MFX that was designed for a larger number of patients. Due to logistical challenges and lack of patients, the study was not completed and the preliminary results have not been published. Exclusion criteria were: radiographic evidence of OA or evidence of cartilage degeneration at arthroscopy; patients with concomitant disease, injury, or malalignment. The group included 10 men and 5 women (1 woman had bilateral injuries) with a median age at index operation of 37 years (30–47). The mean size of the traumatic chondral injury was 261 mm^2^ (120–600) (Table). Patients were randomized to either MFX or ACI treatment. However, 2 patients in each group had to be reoperated within the first 2 years (Figure 1). This resulted in 9 knees finally treated with ACI and 6 knees treated with MFX drilling. One patient initially treated with MFX was reoperated with mosaicoplasty as a salvage procedure. In that patient, cartilage-bone plugs were harvested from unloaded joint regions and implanted to the injury site.

The medical records, including surgical reports and archived radiographs, were studied in all patients until the 17-year follow-up (Figure 3 and Table). dGEMRIC values were compared (Figure 4) to a cohort of 19 asymptomatic individuals (mean age 24 years) that previously had been investigated with an identical MRI protocol (Tiderius et al. 2001).

### Surgical procedures

At the first operation, the cartilage injury was verified with arthroscopy and the treatment randomized. Knees randomized to ACI had cartilage harvested from unloaded cartilage on the upper medial femoral condyle (Brittberg et al. 1994). The donor site was remote from the cartilage lesion. The second surgery, 2–3 weeks later, was a mini-arthrotomy with debridement of the cartilage lesion to stable edges in all patients. The knees randomized to ACI had a periosteal flap sutured over the lesion and sealed by fibrin glue, under which the in-vitro expanded chondrocytes were injected (Brittberg et al. 1994). The knees randomized to MFX had the lesion drilled with a ∅2 mm drill, hole centers spaced 6 mm apart. The protocol for postoperative management of ACI described by Brittberg et al. (1994) was followed for both groups consisting of 6 weeks of unloading followed by 6 weeks of progressive weight-bearing and detailed supervised physiotherapy.

### dGEMRIC

The dGEMRIC investigations were performed on average 2 years after the cartilage repair procedure using a standard 1.5 T MRI system with a dedicated knee coil (Magnetom Vision; Siemens Medical Solutions, Erlangen, Germany). Gd-DTPA^2-^ (Magnevist®, Bayer Schering Pharma AG, Berlin, Germany) at 0.3 mmol/kg body weight dosage was injected intravenously. To optimize the distribution of Gd-DTPA^2-^ into the cartilage, the patients exercised by walking up and down stairs for 10 min, starting 5 min after the injection. Post-contrast MR imaging was performed 2 hours after the injection according to a standardized protocol (Tiderius et al. 2001). Two sagittal slices covering the central parts of the weight-bearing lateral and medial femoral cartilage respectively were acquired using sets of 6 turbo inversion recovery images with different inversion times (TI =50, 100, 200, 400, 800, and 1600 ms), from which the T1 relaxation time was subsequently calculated. Other imaging parameters were: TR =3000 ms, TE =15 ms, turbofactor 7, field of view (FOV) 120 × 120 mm^2^, matrix =256 × 256, slice thickness =3 mm.

**Figure 2.**
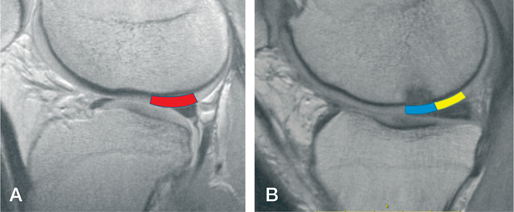
Illustration of how the regions of interest (ROIs) for dGEMRIC were drawn. All ROIs included full-thickness cartilage. In the lateral compartment (A), the ROI was drawn from the center of the tibial plateau to the rear insertion of the meniscus (red), according to a standardized protocol (Tiderius et al. 2004b). In the medial compartment (B), one ROI included the repair tissue (blue) and one ROI (yellow) the remaining weight-bearing cartilage to the rear insertion of the meniscus.

Regions of interest (ROIs) were drawn (Figure 2) in the weight-bearing central parts of the lateral and medial femoral cartilage, respectively. In the medial compartment, 1 ROI covered the area of cartilage repair tissue and 1 ROI was drawn in adjacent, non-injured weight-bearing cartilage. In the lateral compartment, the ROI was drawn between the center of the tibia plateau to the rear insertion of the meniscus according to a previously validated protocol (Tiderius et al. 2004b). Results are presented as mean T1 ms of each ROI (the dGEMRIC index).

### Radiography

At median 17 years (15–19) after the cartilage repair surgery, standardized weight-bearing radiographs in 20° flexion of both knees were obtained. Blinded to clinical presentation and to the type of cartilage repair procedure, assessment of the ante-posterior radiographs was performed by 2 of the authors independently: an orthopedic surgeon specialized in joint replacement (JT) and a senior radiologist specialized in skeletal radiology (BL). In cases of discrepancy the images were reassessed by the 2 investigators together and consensus was reached. The OARSI atlas (Altman and Gold 2007) was used for the medial and lateral compartments respectively, grading radiographic change on a 4-point scale for joint space narrowing (JSN) (0–3, 0 = no evidence of JSN) and marginal osteophytes of femoral and tibial condyles (0–3 each, 0 = no bony change). Dichotomization for diagnosis of radiographic OA was defined as any of the following criteria fulfilled in either of the 2 tibiofemoral compartments: JSN ≥2, osteophyte score ≥2, or JSN grade 1 in combination with osteophyte grade 1 in the same compartment. This definition (Englund et al. 2003) approximates grade 2 knee OA based on the Kellgren–Lawrence scale. 2 patients could not partake in the radiographic examination. However, both these patients had previous visits to an orthopedic surgeon due to knee pain respectively 4 and 9 years postoperatively. Weight-bearing radiographs from these visits demonstrated OA by the above criteria.

## PROMS

Patient-related outcome measures (PROMS) were completed at the 17-year follow-up by self-administered pen on paper forms for VAS, Lysholm, and KOOS. The algorithm based on the KOOS score described by Englund et al. (2003) was used to dichotomize for clinical OA. Patients who had been treated for OA with osteotomy or arthroplasty were excluded from PROMS analysis.

### Statistics

SPSS 25 Statistics for Windows (IBM Corp, Armonk, NY, USA) and SigmaPlot 11.0 (Systat Software, San Jose, CA) was used for statistical analysis. Despite the fact that data from 1 bilateral operation are not independent, we included both knees in that patient for analysis. After testing for normal distribution (Shapiro–Wilk) and equal variance (Levene’s mean test), the Student t-test was used for continuous variables. A paired test was used for regional measurements in the same knee; a non-paired test was used in all other instances, and 2-tailed distribution was assumed in all tests. Spearman’s Rho was used for correlation of ordinal data and continuous variables. Fisher’s exact test was used to compare the distribution in cases of 2 dichotomous variables. The statistical power was low due to the few patients eligible for this study.

### Ethics, funding, and potential conflicts of interest

The study was approved by the ERB at Lund University (Etikprövningsnämnden #EPN:2014/752, LU#73-96 and LU#651-00), the Radiation Protection Committee (Strålskyddskommiten #SSFo2014-050), and the Image Research Committee (BOF053).

The study was supported by grants from the Regional Research Council of Region Skåne, Governmental Funding of Clinical Research within National Health Service (ALF), Erik and Angelica Sparres forskningsstiftelse and the Johan and Greta Kock Foundation. No conflicts of interest declared.

## Results

### OA, radiographic, and symptomatic

**Figure 3.**
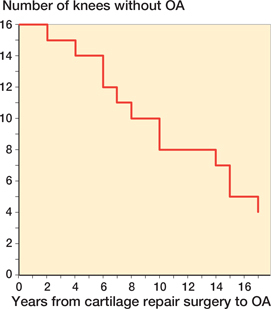
Temporal assessment of OA after surgical repair of a chondral injury on the medial femoral condyle. OA development was defined as either high tibial osteotomy, arthroplasty, or radiographic OA. Time points of OA diagnosis are from surgery date (HTO or joint replacement) or date of radiographic OA either due to radiographic evaluation of clinical symptoms in the intermediate time or by radiographs at the 17-year follow-up. The initial number of knees (n = 16) without OA already starts to decrease two years after surgery. At the end of the study period (17 years), only 4 knees lack radiographic OA.

All 16 knees could be assessed for radiographic OA (rOA) after mean 17 years follow-up (Figure 1 and Table). 6 knees had received OA surgery; 2 had high tibial osteotomy, 2 had unicompartmental medial knee arthroplasty and 2 had total knee arthroplasty. 6 knees had radiographic OA based on OARSI scores, and 4 knees had no radiographic OA. Thus 12 of 16 knees had failed by progressing to OA (Figure 3).

The KOOS score was indicative of OA (Englund et el. 2003) in all but 2 knees with a normal age-matched KOOS score and no radiographic OA (Table). All 4 knees that needed a second cartilage repair procedure progressed to OA. All MFX-treated knees and 6 of 10 ACI knees developed radiographic OA (p = 0.2).

### dGEMRIC

All knees were examined with dGEMRIC at median 27 (12–57) months after the cartilage repair procedure. The 4 early graft failure reoperations were performed 12–15 months prior to the dGEMRIC examination.

**Figure 4.**
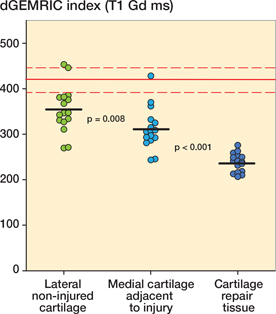
dGEMRIC index of the 3 investigated ROIs in each knee (n = 16): the lateral femoral condyle (354 ms SD 51), the medial cartilage adjacent to the cartilage lesion (312 ms, SD 46) and the repair tissue (237 ms, SD 20). The dGEMRIC index was lower in repair tissue vs. adjacent cartilage in the medial femoral cartilage (p < 0.001). The dGEMRIC index was higher in the uninjured lateral femoral cartilage than in the medial cartilage adjacent to the cartilage lesion (p < 0.008). Horizontal black bars are mean values. For comparison, the red solid (mean) and dashed lines (SD) represent the dGEMRIC index in healthy volunteers previously investigated with an identical protocol by our group (Tiderius et al. 2001).

The mean dGEMRIC index (T1Gd in ms) differed between the 3 ROIs as illustrated in Figure 4. The mean dGEMRIC index was 33% lower in the repair tissue compared with the non-affected lateral femoral cartilage, 237 vs. 354 ms (p < 0.001). In addition, the dGEMRIC index in the cartilage adjacent to the lesion was lower than in the non-affected lateral femoral cartilage, 311 ms (SD 58) vs. 354 ms (SD 51) (p = 0.008). The cartilage adjacent to the repair tissue was higher after ACI compared with MFX surgery with borderline significance: 331 ms (SD 47) vs. 283 ms (SD 33) (p = 0.05).

**Figure 5.**
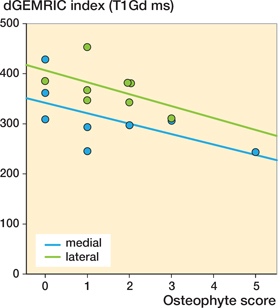
The dGEMRIC index of medial adjacent cartilage (blue) correlated negatively with radiographic osteophyte score at 17 years, r = –0.75 (p = 0.03). A similar trend, but not statistically significant, was found in the lateral compartment (green), r = –0.60 (p = 0.1). Note that the 6 knees that had already had surgery for OA (HTO or arthroplasty) were excluded from this correlation analysis as were the 2 knees that had only early follow-up radiographs.

The dGEMRIC index in the medial cartilage adjacent to the lesion correlated negatively with the radiographic osteophyte score in the medial compartment at the 17-year follow-up, r = –0.75 (p = 0.03). A similar trend, although not statistically significant, was found in the lateral compartment, r = –0.60 (p = 0.1) (Figure 5). JSN did not seem to correlate in the medial compartment r = –0.25 (p = 0.5); in the lateral compartment there was no correlation as all knees had zero JSN.

There was a trend towards higher dGEMRIC index in the adjacent cartilage of the 4 knees that did not develop radiographic OA compared with OA knees, 348 (SD 61) vs. 300 (SD 35) (p = 0.07).

BMI, sex, and size of injury were similar between knees that developed radiographic OA and those that did not. Non-OA patients were on average 4 years younger than patients who developed radiographic OA (p = 0.1).

## Discussion

The main finding of this study is that most (12/16) of patients that had surgical treatment for a traumatic chondral injury on the medial femoral condyle had developed radiographic OA 17 years after the surgery. In addition, 2 of the 4 patients with no radiographic OA had clinical OA according to the KOOS score. For comparison, the prevalence of radiographic knee OA in the general population of similar age (54–65 years) is between 10% and 23% (Felson et al. 1987, Turkiewicz et al. 2015).

The goal of cartilage repair is twofold: to alleviate symptoms and to avoid future OA. Despite decades of research there is no consensus regarding the optimal treatment for traumatic cartilage injuries. In a short- and mid-term perspective, several studies of MFX report good results with a large proportion of patients returning to high levels of activity (Mithoefer et al. 2009, Erggelet and Vavken 2016). However, 10–15 years after the operation, results deteriorate with half of patients having radiographic OA (Gobbi et al. 2014, Knutsen et al. 2016). Theoretically, the reason for failure might be explained by the fact that the repair cartilage after MFX operation lacks collagen II and does not show the zonal organization of hyaline cartilage (Mithoefer et al. 2009, Erggelet and Vavken 2016). By contrast, the ACI technique was designed to yield repair tissue with a hyaline-like structure that potentially also has mechanical properties that resembles healthy cartilage. A recent review of 9 ACI studies (Pareek et al. 2016) with 9–13 years’ follow-up reported on average 81% successful results, defined by no diagnosed graft failures and good or excellent clinical results. However, the only study that presented radiographic follow-up (Martinčič et al. 2014) at 10 years postoperatively found OA in half of the cases, i.e., similar to that reported for MFX.

An RCT of ACI vs. MFX with a 15-year follow-up of 78 knees (Knutsen et al. 2016) had one-third failures and half of the remaining knees had radiographic OA with no difference between the treatment groups. The small numbers of patients in our study, in combination with the crossover that occurred, hampers a relevant comparison between MFX and ACI treatments. However, it should be pointed out that the 4 patients that did not end up with radiographic OA were all treated with ACI. In addition, dGEMRIC indicated better cartilage quality in the cartilage adjacent to the repair tissue in ACI compared with MFX patients. The evaluation of cartilage quality a few years after the cartilage repair procedure is a major strength of our study. Obviously, the assessment of cartilage status is equally relevant for MFX and ACI cases.dGEMRIC is a validated in-vivo technique to estimate cartilage quality, in particular the glucosaminoglycan content. We found a low dGEMRIC index in the repair tissue both after MFX and ACI indicating fibrocartilage with low GAG content.

Several previous studies have shown that a low dGEMRIC index is associated with an increased risk of future radiographic OA, both in the hip (Cunningham et al. 2006, Palmer et al. 2017) and in the knee (Owman et al. 2008, 2014). For example, in middle-aged patients (mean age 50 years) with superficial cartilage fibrillation on the femoral cartilage, a low dGEMRIC index (circa 300 ms) was associated with radiographic OA in two-thirds of patients 6 years after the dGEMRIC investigation (Owman et al. 2008).dGEMRIC as a prognostic tool was suggested also in our study; we found a correlation between a low dGEMRIC index in the cartilage adjacent to the repair tissue and the future prevalence of radiographic OA. This may indicate that the surrounding cartilage should be evaluated at the time of cartilage repair surgery. In support, a clinical MFX study found that visual mild degeneration of surrounding cartilage at the primary operation had a worse outcome at 10–14 years’ follow-up than patients with normal-appearing cartilage (Solheim et al. 2016). Importantly, in our study, we do not know if low cartilage quality was present already at the time of surgery, or if it developed between surgery and the dGEMRIC investigation, approximately 2 years later.

Also, the non-injured lateral cartilage demonstrated lower dGEMRIC values (Figure 4) than previously observed in healthy volunteers (Tiderius et al. 2001) investigated with an identical protocol. This finding may reflect that cartilage degeneration in the medial compartment affects the whole joint, with GAG loss also in the lateral femoral condyle. Other possible explanations for this finding are reduced loading during rehabilitation as the dGEMRIC index is known to respond to changes in activity level (Roos and Dahlberg 2005), correlate to level of activity (Tiderius et al. 2004a) and correlate to thigh muscle strength (Ericsson et al. 2009).

A strength of our study is that all included patients could be assessed regarding OA development and that the study had a strict inclusion criterion: an isolated traumatic chondral injury only on the medial femoral condyle. The main limitation is the small number of patients, resulting in low statistical power, especially regarding the comparison between the 2 surgical methods, MFX and ACI.

Furthermore, the value of PROMS was limited because several patients had major joint surgery between the cartilage repair surgery and the 17-year follow-up.

In summary, we found a high prevalence of OA at follow-up 17 years after cartilage repair. There was no evidence of hyaline-like cartilage 2 years after ACI, as demonstrated with a low dGEMRIC index. The negative correlation between the dGEMRIC index in the adjacent cartilage and future OA indicates that dGEMRIC can predict future radiographic OA and that the quality of the surrounding cartilage influences the outcome after cartilage repair surgery.

The authors would like to thank Håkan Lövkvist at the Department of Medical Statistics and Epidemiology at Lund University for statistical advice.

Study design: JT, LD and CT. Analysis of radiographs: JT and BL. Analysis of MR images: JT, JS and CT. Collection of clinical data: JT. Writing of manuscript: JT, PN, JS, LD and CT. Revision of manuscript: JT and CT.

*Acta* thanks Martin Lind and other anonymous reviewers for help with peer review of this study.
